# The decreased SIRT1 level may account for the lipid profile in chronic kidney disease

**DOI:** 10.1186/s40709-019-0101-2

**Published:** 2019-10-16

**Authors:** Gang Chen, Xuemei Li

**Affiliations:** Dept. of Nephrology, Peking Union Medical College Hospital, Peking Union Medical College, Chinese Academy of Medical Science, 100730 Beijing, China

**Keywords:** Silent information regulator-1, Chronic kidney disease, Dyslipidemia, Sterol regulatory element-binding proteins

## Abstract

Dysregulated lipid profile with hypertriglyceridemia and increased low-density lipoprotein (LDL) is common in chronic kidney disease (CKD) whereas the reason is unclear. A similar phenomenon is found in the elder population. Silent information regulator-1 (SIRT1) associates with many modulators regulating lipid metabolism and results in increased expression of sterol regulatory element-binding proteins (SREBPs), which functions as a key modulator in lipid synthesis. Since CKD is being viewed as a premature aging model and SIRT1 is known to decrease during the process of aging, we hypothesize that SIRT1 level is reduced in the liver when CKD develops and eventually result in dysregulated lipid profile.

## Background

Chronic kidney disease (CKD) impacts 11–13% of general population [1]. Dyslipidemia is common in CKD and it develops during the early stages of CKD [2]. The primary finding of lipid profiles in CKD patients is hypertriglyceridemia [3]. Epidemiological studies also revealed the increased LDL cholesterol in CKD [4, 5]. The similar lipid profile is characterized in the elder population [6] and is associated with increased risk of myocardial infarction, ischemic heart disease, and death [7]. So far, the preponderance of hypertriglyceridemia and elevated LDL in CKD has not been well elucidated.

CKD is increasingly being viewed as a premature aging model [8, 9]. The uremic phenotype is characterized by many features of aging, such as atherosclerosis, osteoporosis, inflammation, oxidative stress, insulin resistance, frailty, skin atrophy, and cognitive dysfunction [10, 11]. It is not surprising to find the similarity in the lipid profile in both CKD and elder population.

## Hypothesis

The circulating lipid levels are determined by the balance between their synthesis and clearance. The synthesis of triglyceride (TG) and cholesterols are regulated by a common family of sterol regulatory element-binding proteins (SREBPs): SREBP-1a, SREBP-1c, and SREBP-2. SREBP-1c is the predominant isoform in human liver and is the key modulator of TG synthesis [12]. De novo synthesis of TG from dietary carbohydrates occurs in the liver, where TG is packaged as very-low-density lipoprotein cholesterol. SREBP-1c regulates TG and fatty acid synthesis, while peroxisome proliferator-activated receptor-α (PPARα), another lipid modulator, stimulates fatty acid β-oxidation. Increased SREBP-1c and decreased PPARα promote net TG synthesis. Meanwhile, lipases hydrolyze TG, serve them as an energy source or store them in adipose tissues, therefore clear TG from circulation. Cholesterol biosynthesis is mainly regulated by SREBP-2, which is also highly expressed in the liver [13]. Mature SREBP-2 modulates 3-hydroxy-3-methylglutaryl-CoA reductase (HMGCR), the rate-limiting enzyme in cholesterol synthesis [14, 15]. Cholesterol converts into bile acids (BAs) in the mammalian liver while cholesterol 7 α-hydroxylase (CYP7A1) catalyzes at the rate-controlling step in this process [16]. BAs are important metabolic regulators and signaling molecules that control lipid homeostasis [17, 18]. Two explanations exist regarding the relationship between BAs and lipid synthesis. At the metabolite level, BAs synthesis reduces hepatic cholesterol/oxysterol levels and stimulates de novo cholesterol synthesis to provide substrates for CYP7A1 [17]. Decreased intracellular oxysterol level stimulate SREBPs and by whose activation, TG and cholesterol synthesis are stimulated. At the transcriptional level, BAs can stimulate the farnesoid X receptor (FXR) and induce the expression of short heterodimer partner (SHP). SHP then inhibits the activity of liver X receptor (LXR) and eventually represses SREBPs and CYP7A1 expression [19, 20]. Furthermore, liver-intestine crosstalk via fibroblast growth factor 15/19 (FGF15/19) exists. BAs stimulate the intestine expression of FGF15/19. FGF15/19 acts on hepatocytes and represses CYP7A1 [21] and SREBPs [22], resulting in decreased BAs and lipid synthesis.

In CKD animal models, there is evidence for increased expression of SREBPs in liver tissue [13, 23], which accounts for increased TG and cholesterol. However, the higher SREBPs expression is not driven by elevated BAs synthesis in CKD. Several studies have reported that hepatic CYP7A1 activity is virtually identical in chronic renal failure (CRF) rats and healthy controls [24–26], and BAs production rate is intact as well [26]. Since the contribution of the CYP7A1 pathway to stimulate SREBPs in CKD appears to be minor, the FXR-SHP-SREBPs regulatory cascade may be an important mechanism linking BAs and lipid synthesis in CKD. FXR agonist GW4064 can decrease lipid levels while *Fxr*^−*/*−^ mice show decreased expression of SHP and increased lipid levels in both serum and liver [27]. Increased SREBPs expression in CKD may due to decreased FXR-SHP activity.

Silent information regulator-1 (SIRT1) is one of the mammals dominant sirtuins, which are a group of NAD-dependent deacetylases linked to cellular energy metabolism and the redox state through multiple signaling and survival pathways. SIRT1 is decreased in both transcriptional and posttranscriptional conditions during aging [28]. It is related to longevity, cellular protection, regulation of energy metabolism [29], as well as lipids metabolism [30]. In the kidney, SIRT1 may inhibit renal cell apoptosis, inflammation, and fibrosis [31]. The decreased kidney SIRT1 level is associated with deteriorated kidney function,and the upregulation of SIRT1 demonstrates renoprotection [32–34]. Diabetic nephropathy is one of the leading causes of CKD, in which the role of SIRT1 has been more intensively studied. Diabetic rat model showed lower renal SIRT1 expression and less creatinine clearance rate [35]. Serum SIRT1 levels in diabetic nephropathy patients are also decreased significantly [36]. However, SIRT1 levels in organs other than kidney in CKD condition has not been well investigated. We hypothesize that SIRT1 level is reduced in liver when CKD develops and eventually result in altered lipid profile.

Researchers have revealed the influence of SIRT1 on various important modulators in lipid regulation. SIRT1 deacetylates SREBP-1 and SREBP-2 and then destabilizes their activities [30, 37], resulting in decreased TG and cholesterol synthesis. SIRT1 can directly deacetylate FXR and the down-regulation of hepatic SIRT1 increases FXR acetylation, which inhibits its transcriptional activity [38, 39]. SIRT1 also interacts with other modulators in lipid metabolism, including LXR, PPARα, and PPARγ coactivator 1α (PGC-1α), a transcription co-factor known to activate PPARα [40, 41]. SIRT1 deacetylates LXR and subsequently promotes its degradation upon ligand binding [42]. Evidence showed that *Sirt1* knockout animals have increased hepatic LXR protein [42]. SIRT1 activates PGC-1α and consequently stimulates PPARα, therefore facilitates fatty acid β-oxidation [40]. Increased LXR and decreased PPARα levels result in a higher level of lipids. Hepatocyte-specific deletion of *Sirt1* in mice demonstrated altered fatty acid and lipid metabolism which led to hepatic steatosis [43].

Furthermore, SIRT1 deacetylates transcription factors forkhead box O (FOXO), which also downregulates cholesterol synthesis. FOXO1 can specifically recognize insulin response element (IRE) sequence in the SREBP-2 promoter region, and negatively regulate the transcriptional level of SREBP-2 [14]. *Foxo1* knockout mice were observed with increased expression of fatty acids and LDL [44]. A recent study also unveiled its regulatory factor in BAs metabolism. Liver-specific *Foxo1* knockout mice revealed that FOXO1 is required for expression of a subset of BAs synthesis genes and loss of hepatic *Foxo1* results in an unusual BAs profile that impairs the ability of FXR to reduce TG and cholesterol [45].

Stimulating SIRT1 level can ameliorate lipid profiles. Nicotinic acid, one precursor of NAD + which increases cellular NAD + levels and SIRT1 activity [46], has been used as a lipid-lowering regimen [47]. Calorie restriction (CR) is another way to boost SIRT1. CR delays the onset of various aging-related metabolic and cardiovascular disorders [48] and it is proven to lower TG and cholesterol level in a human study [49].

## Conclusion

**Fig. 1:**
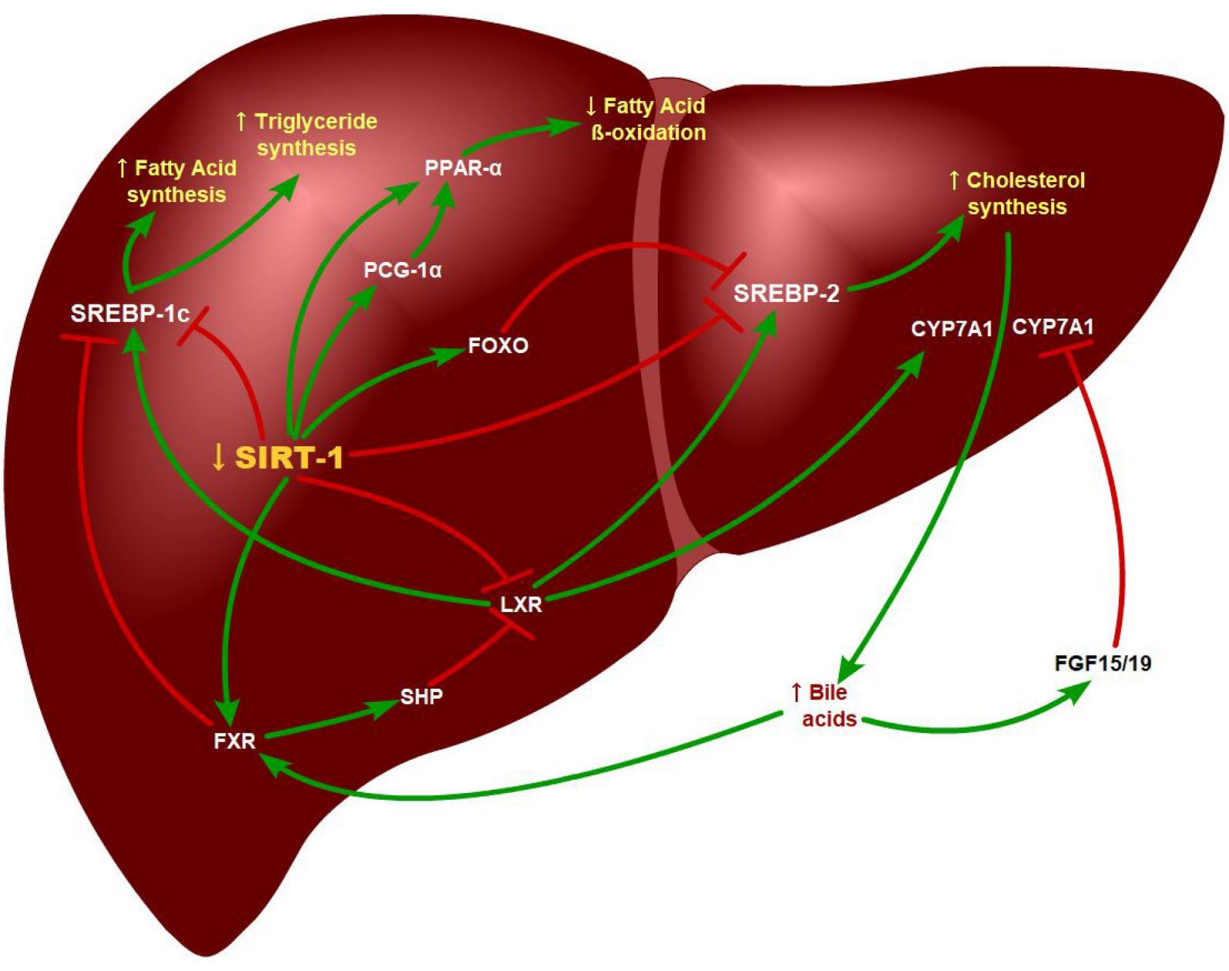
The key pathways and target genes of the SIRT1 concerning cholesterol, fatty acids, bile acid, and triglyceride homeostasis. Decreased SIRT1 eventually promotes triglyceride, fatty acid, and cholesterol synthesis, whereas represses fatty acid beta-oxidation. As a result, the lipid profiles alter due to the SIRT1 level

In conclusion, we hypothesize that SIRT1 plays an important role in lipid metabolism and that decreased SIRT1 level would result in altered lipid profile in CKD. During aging, SIRT1 level decreases in different tissues. Since CKD is being viewed as an aging model, our hypothesis is that tissue SIRT1 level is reduced in the liver when CKD develops, and subsequently disrupts the expression of PPARα, PGC1α, FXR, LXR, and FOXO, increase the expression of SREBPs, and eventually result in altered lipids profiles. Insufficient expression of FXR impairs FXR-SHP pathway in the liver, as well as FXR-FGF15/19 feedback in the intestine. And therefore, BAs synthesis is not repressed by FXR-SHP-CYP7A1 cascade in CKD while dyslipidemia is presented (Fig.1).

## How to evaluate this hypothesis

To evaluate this hypothesis, the most important link would be the findings of decreased SIRT1 level in CKD. First, we will investigate the CKD mice models. By setting 5/6 kidney nephrectomy and adenine-induced CKD models, we will compare SIRT1 mRNA and protein levels in different tissues between CKD mice and healthy controls. The downstream lipid modulators of SIRT1 and lipid profiles would also be compared. If the reverse correlation of SIRT1 and renal function would be revealed, we will further verify it in human studies. A study can be designed by including patients undergoing liver surgeries with matched age and sex and then correlate serum/tissue SIRT1 levels with serum creatinine. If serum SIRT1 levels were positively correlated with hepatic ones whereas both SIRT1 levels negatively correlated with creatinine, we will take serum SIRT1 levels as surrogates to indicate hepatic SIRT1 and finally compare serum SIRT1 levels and lipid profiles in early and advanced CKD patients.

## References

[1] Hill NR, Fatoba ST, Oke JL, Hirst JA, O’Callaghan CA, Lasserson DS (2016). Global prevalence of chronic kidney disease—a systematic review and meta-analysis. PLoS ONE.

[2] Harper CR, Jacobson TA (2008). Managing dyslipidemia in chronic kidney disease. J Am Coll Cardiol.

[3] Ritz E, Wanner C (2006). Lipid changes and statins in chronic renal insufficiency. J Am Soc Nephrol.

[4] Vaziri ND (2010). Lipotoxicity and impaired high density lipoprotein-mediated reverse cholesterol transport in chronic kidney disease. J Ren Nutr..

[5] Schaeffner ES, Kurth T, Curhan GC, Glynn RJ, Rexrode KM, Baigent C (2003). Cholesterol and the risk of renal dysfunction in apparently healthy men. J Am Soc Nephrol.

[6] Windler E, Schöffauer M, Zyriax BC (2007). The significance of low HDL-cholesterol levels in an ageing society at increased risk for cardiovascular disease. Diab Vasc Dis Res..

[7] Nordestgaard BG, Benn M, Schnohr P, Tybjaerg-Hansen A (2007). Nonfasting triglycerides and risk of myocardial infarction, ischemic heart disease, and death in men and women. JAMA.

[8] Thang OH, Serné EH, Grooteman MP, Smulders YM, Ter Wee PM, Tangelder GJ (2012). Premature aging of the microcirculation in patients with advanced chronic kidney disease. Nephron Extra..

[9] Bian A, Neyra JA, Zhan M, Hu MC (2015). *Klotho*, stem cells, and aging. Clin Interv Aging.

[10] Stenvinkel P, Larsson TE (2013). Chronic kidney disease: a clinical model of premature aging. Am J Kidney Dis.

[11] Kooman JP, Kotanko P, Schols AM, Shiels PG, Stenvinkel P (2014). Chronic kidney disease and premature ageing. Nat Rev Nephrol..

[12] Eberlé D, Hegarty B, Bossard P, Ferre P, Foufelle F (2004). SREBP transcription factors: master regulators of lipid homeostasis. Biochimie.

[13] Chmielewski M, Sucajtys-Szulc E, Kossowska E, Swierczynski J, Rutkowski B, Boguslawski W (2007). Increased gene expression of liver SREBP-2 in experimental chronic renal failure. Atherosclerosis..

[14] Li Y, Wu S (2018). Epigallocatechin gallate suppresses hepatic cholesterol synthesis by targeting SREBP-2 through SIRT1/FOXO1 signaling pathway. Mol Cell Biochem.

[15] Tao R, Xiong X, DePinho RA, Deng CX, Dong XC (2013). Hepatic SREBP-2 and cholesterol biosynthesis are regulated by FoxO3 and Sirt6. J Lipid Res.

[16] Chiang JY (2009). Bile acids: regulation of synthesis. J Lipid Res.

[17] Chiang JY (2013). Bile acid metabolism and signaling. Compr Physiol..

[18] Hofmann AF, Hagey LR (2014). Key discoveries in bile acid chemistry and biology and their clinical applications: history of the last eight decades. J Lipid Res.

[19] Watanabe M, Houten SM, Wang L, Moschetta A, Mangelsdorf DJ, Heyman RA (2004). Bile acids lower triglyceride levels via a pathway involving FXR, SHP, and SREBP-1c. J Clin Invest..

[20] Kalaany NY, Mangelsdorf DJ (2006). LXRS and FXR: the yin and yang of cholesterol and fat metabolism. Annu Rev Physiol.

[21] Inagaki T, Choi M, Moschetta A, Peng L, Cummins CL, McDonald JG (2005). Fibroblast growth factor 15 functions as an enterohepatic signal to regulate bile acid homeostasis. Cell Metab.

[22] Bhatnagar S, Damron HA, Hillgartner FB (2009). Fibroblast growth factor-19, a novel factor that inhibits hepatic fatty acid synthesis. J Biol Chem.

[23] Korczynska J, Stelmanska E, Nogalska A, Szolkiewicz M, Goyke E, Swierczynski J (2004). Upregulation of lipogenic enzymes genes expression in white adipose tissue of rats with chronic renal failure is associated with higher level of sterol regulatory element binding protein-1. Metabolism..

[24] Vaziri ND, Sato T, Liang K (2003). Molecular mechanisms of altered cholesterol metabolism in rats with spontaneous focal glomerulosclerosis. Kidney Int.

[25] Gai Z, Chu L, Hiller C, Arsenijevic D, Penno CA, Montani JP (2014). Effect of chronic renal failure on the hepatic, intestinal, and renal expression of bile acid transporters. Am J Physiol Renal Physiol.

[26] Liang K, Vaziri ND (1997). Gene expression of LDL receptor, HMG-CoA reductase, and cholesterol-7a-hydroxylase in chronic renal failure. Nephrol Dial Transplant.

[27] Sinal CJ, Tohkin M, Miyata M, Ward JM, Lambert G, Gonzalez FJ (2000). Targeted disruption of the nuclear receptor FXR/BAR impairs bile acid and lipid homeostasis. Cell.

[28] Yuan Y, Cruzat VF, Newsholme P, Cheng J, Chen Y, Lu Y (2016). Regulation of SIRT1 in aging: roles in mitochondrial function and biogenesis. Mech Ageing Dev.

[29] Bordone L, Guarente L (2005). Calorie restriction, SIRT1 and metabolism: understanding longevity. Nat Rev Mol Cell Biol.

[30] Ponugoti B, Kim DH, Xiao Z, Smith Z, Miao J, Zang M (2010). SIRT1 deacetylates and inhibits SREBP-1C activity in regulation of hepatic lipid metabolism. J Biol Chem.

[31] Dong YJ, Liu N, Xiao Z, Sun T, Wu SH, Sun WX (2014). Renal protective effect of sirtuin 1. J Diabetes Res..

[32] Jung YJ, Lee JE, Lee AS, Kang KP, Lee S, Park SK (2012). SIRT1 overexpression decreases cisplatin-induced acetylation of NF-κB p65 subunit and cytotoxicity in renal proximal tubule cells. Biochem Biophys Res Commun.

[33] Hasegawa K, Wakino S, Yoshioka K, Tatematsu S, Hara Y, Minakuchi H (2010). Kidney-specific overexpression of Sirt1 protects against acute kidney injury by retaining peroxisome function. J Biol Chem.

[34] Hao CM, Haase VH (2010). Sirtuins and their relevance to the kidney. J Am Soc Nephrol.

[35] Kitada M, Takeda A, Nagai T, Ito H, Kanasaki K, Koya D (2011). Dietary restriction ameliorates diabetic nephropathy through anti-inflammatory effects and regulation of the autophagy via restoration of Sirt1 in diabetic Wistar fatty (fa/fa) rats: a model of type 2 diabetes. Exp Diabetes Res..

[36] Shao Y, Ren H, Lv C, Ma X, Wu C, Wang Q (2017). Changes of serum Mir-217 and the correlation with the severity in type 2 diabetes patients with different stages of diabetic kidney disease. Endocrine.

[37] Walker AK, Yang F, Jiang K, Ji JY, Watts JL, Purushotham A (2010). Conserved role of SIRT1 orthologs in fasting-dependent inhibition of the lipid/cholesterol regulator SREBP. Genes Dev.

[38] Kemper JK, Xiao Z, Ponugoti B, Miao J, Fang S, Kanamaluru D (2009). FXR acetylation is normally dynamically regulated by p300 and SIRT1 but constitutively elevated in metabolic disease states. Cell Metab.

[39] Kazgan N, Metukuri MR, Purushotham A, Lu J, Rao A, Lee S (2014). Intestine-specific deletion of SIRT1 in mice impairs DCoH2-HNF-1α-FXR signaling and alters systemic bile acid homeostasis. Gastroenterology.

[40] Purushotham A, Schug TT, Xu Q, Surapureddi S, Guo X, Li X (2009). Hepatocyte-specific deletion of SIRT1 alters fatty acid metabolism and results in hepatic steatosis and inflammation. Cell Metab.

[41] Mori Y, Hirano T, Nagashima M, Shiraishi Y, Fukui T, Adachi M (2007). Decreased peroxisome proliferator-activated receptor alpha gene expression is associated with dyslipidemia in a rat model of chronic renal failure. Metabolism..

[42] Li X, Zhang S, Blander G, Tse JG, Krieger M, Guarente L (2007). SIRT1 deacetylates and positively regulates the nuclear receptor LXR. Mol Cell.

[43] Yin H, Hu M, Liang X, Ajmo JM, Li X, Bataller R (2014). Deletion of SIRT1 from hepatocytes in mice disrupts lipin-1 signaling and aggravates alcoholic fatty liver. Gastroenterology.

[44] Li K, Zhang J, Yu J, Liu B, Guo Y, Deng J (2015). MicroRNA-214 suppresses gluconeogenesis by targeting activating transcriptional factor 4. J Biol Chem.

[45] Haeusler RA, Pratt-Hyatt M, Welch CL, Klaassen CD, Accili D (2012). Impaired generation of 12-hydroxylated bile acids links hepatic insulin signaling with dyslipidemia. Cell Metab.

[46] Bogan KL, Brenner C (2008). Nicotinic acid, nicotinamide, and nicotinamide riboside: a molecular evaluation of NAD + precursor vitamins in human nutrition. Annu Rev Nutr.

[47] Carlson LA (2005). Nicotinic acid: the broad-spectrum lipid drug. A 50th anniversary review. J Intern Med.

[48] Most J, Tosti V, Redman LM, Fontana L (2017). Calorie restriction in humans: an update. Ageing Res Rev..

[49] Collet TH, Sonoyama T, Henning E, Keogh JM, Ingram B, Kelway S (2017). A Metabolomic Signature of Acute Caloric Restriction. J Clin Endocrinol Metab.

